# Ni–Fe Dual‐Site Polymer Catalyst for High Performance and Stable Electrochemical Urea Synthesis from CO_2_ and NO_3_^−^

**DOI:** 10.1002/cssc.202502676

**Published:** 2026-03-12

**Authors:** Daming Feng, Zhenghao Lyu, Qian Zhang, Hui Li, Fengxia Wei, Zhenglong Li, Hongge Pan, Tianyi Ma

**Affiliations:** ^1^ College of Chemistry Liaoning University Shenyang China; ^2^ State Key Laboratory of Biobased Transportation Fuel Technology Zhejiang University Hangzhou China; ^3^ Centre for Atomaterials and Nanomanufacturing (CAN) School of Science RMIT University Melbourne Australia; ^4^ ARC Industrial Transformation Research Hub for Intelligent Energy Efficiency in Future Protected Cropping (E2Crop) Melbourne Australia; ^5^ Mark Wainwright Analytical Center University of New South Wales Sydney Australia; ^6^ Institute of Science and Technology for New Energy Xi’an Technological University Xi’an China

**Keywords:** C–N bond formation, CO_2_ utilization, electrochemical urea synthesis, Ni–Fe bimetallic catalyst, nitrate reduction

## Abstract

The electrochemical synthesis of urea from nitrate (NO_3_^−^) and carbon dioxide (CO_2_) presents a sustainable alternative to conventional methods, mitigating pollution and reducing energy consumption. Herein, a rationally designed Ni–Fe bimetallic pyromellitic acid polymer catalyst (Ni‐PMDA@Fe) is developed for efficient urea electrosynthesis. This metal–organic polymer provides structural robustness, abundant active sites, and a tunable coordination environment, optimizing C–N coupling kinetics. Ni‐PMDA@Fe achieves a urea yield of 449.56 mg h^−1^ g_cat_^−1^ and a Faradaic efficiency (FE) of 41.06% at –0.5 V_RHE_, significantly surpassing monometallic controls (Ni‐BDC, Ni‐PMDA). Fe incorporation modulates the electronic structure of Ni, enhances charge transfer, and stabilizes key reaction intermediates, enabling synergistic NO_3_^−^/CO_2_ coupling. Comprehensive characterization confirms homogeneous Fe doping and a dual‐metal‐site configuration. Unlike single‐atom or monometallic systems, the Ni–Fe dual‐site architecture optimally tunes the adsorption kinetics of critical intermediates. The catalyst maintains a FE exceeding 30% over a 30 h stability test, demonstrating robust operational stability. Furthermore, techno‐economic analysis (TEA) indicates competitive production costs when powered by renewable energy, highlighting scalability potential. This word demonstrates a practical pathway for sustainable urea synthesis by converting pollutants (NO_3_^−^/CO_2_) into value‐added product, thereby contributing to decarbonizing fertilizer production and mitigating nitrogen pollution.

## Graphical abstract

A schematic illustration of the Ni‐PMDA@Fe bimetallic polymer catalyst for electrocatalytic urea synthesis from NO_3_^‐^ and CO_2_. The Fe‐modulated coordination environment enhances active site dispersion and electron transfer efficiency, enabling efficient C–N coupling. Electrochemical performance data and techno‐economic analysis (TEA) confirm the sustainability and scalability of this green urea production strategy.

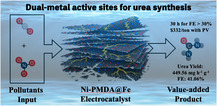

## 1 Introduction

Urea plays a critical role in both industrial chemical production and agricultural fertilization. However, its traditional synthesis via the Bosch–Meiser process is highly energy‐intensive, accounting for 2% of global energy use and over 200 million tons of annual CO_2_ emissions [1, 2, 3, 4, 5]. In contrast, electrosynthesis offers a sustainable alternative by efficiently converting carbon and nitrogen compounds into valuable chemicals using clean energy, thereby addressing environmental concerns [6, 7, 8]. The electrosynthesis of urea involves the co‐reduction of CO_2_ and nitrogen‐containing molecules, such as N_2_, NO, NO_2_^−^, and NO_3_^−^, leveraging electrical energy to form stable C–N bonds. Abundance in the atmosphere, dinitrogen gas is claimed to be one of the competing candidates in urea electrosynthesis, yet hindered by the inertness of N≡N (941 kJ/mol) bonds. The thermodynamic challenges presented by the inertness of N_2_ are further exacerbated by its low solubility and diffusivity in the electrolyte, which impedes reaction kinetics [9, 10, 11, 12, 13]. Alternatively, NO_*x*_ species, such as nitric oxide (NO) and nitrate/nitrite ions (NO_3_^−^/NO_2_^−^), serve as more reactive nitrogen sources with robust activity under reduction reaction conditions. Meanwhile, industrial wastewater contains large amounts of NO_3_^−^/NO_2_^−^ can lead to soil hardening, groundwater contamination, and severe harm to terrestrial and aquatic ecosystems, which urgently requires efficient treatment. Furthermore, researchers have noted that CO_2_ and NO_x_ reduction reactions share similar redox potentials and reaction environments. Particularly, nitrate (NO_3_^−^), being a less stable and more reactive nitrogen source, can be derived from industrial wastewater and nonthermal plasma activation of nitrogen [14, 15]. In this regard, the utilization of NO_3_^−^ and CO_2_ in the electrochemical synthesis of urea is a promising strategy that has become a focal point of current research [16, 17, 18, 19, 20, 21, 22, 23].

Throughout the mechanism of electrochemical synthesis of urea by co‐reduction of NO_3_^−^ and CO_2_, the adsorption and activation of the substrates and the C–N coupling are very important for the generation of urea in one pot [24, 25]. With the aid of high‐performance electrocatalysts, these processes can be promoted, thus facilitating the synthesis of urea. Therefore, efforts have been made in the development of diverse functional electrocatalysts with the purpose of achieving highly efficient urea synthesis. The design of catalysts is pivotal to the efficiency and selectivity of urea electrosynthesis, a process integral to carbon neutrality and green chemistry. Precious metals (Au, Pd, Ru, Ag) are known for their high electrocatalytic efficiency, but their limited availability and high cost pose significant barriers to large‐scale application [26, 27, 28, 29, 30]. Transition metal catalysts (Cu, Co, Ni, Fe) present a more cost‐effective and abundant alternative, making them commercially more viable. Among these, nickel‐based catalysts and iron‐based catalysts have been the subject of extensive research in recent years [31, 32, 33, 34, 35]. He et al. exemplified this by successfully employing NO_3_^−^ and CO_2_ for urea electrosynthesis, achieving a Faradaic efficiency (FE) of 15.6% and 204.2 μg mg^−1^ h^−1^ in a gas‐tight H‐type cell under ambient environment [36]. Likewise, Zhang et al. reported a diatomic catalyst with bonded Fe–Ni pairs can significantly improve the efficiency of electrochemical urea synthesis, achieving a high urea yield rate of 20.2 mmol h^−1^ with corresponding FE of 17.8% [37].

In recent years, scholars have conducted extensive research on design strategies for nickel metal catalysts and iron metal catalysts, exploring various approaches such as single‐atom strategies [38], bimetallic multisite strategies [39], precursor–template coating strategies [40], and metal–organic polymers (MOPs) systems [41]. MOPs systems, in particular, are gaining significant attention for their diverse components, tunable metal valence states, structural variety, and modifiability[42, 43]. These characteristics render MOPs highly applicable in catalytic fields such as hydrogen evolution reaction (HER), carbon dioxide reduction reaction (CO_2_RR), and nitrogen reduction reaction (NRR) [44]. For example, Gao et al. synthesized copper‐based metal–organic framework materials, Cu(III)‐HHTP and Cu(II)‐HHTP, using coordination strategies and oxidizing agents for urea electrosynthesis. The vacant Cu‐3d orbitals in Cu(III)‐HHTP were found to modulate the electronic state, significantly reducing the reaction energy barrier and enhancing C–N coupling efficiency, achieving a maximum yield of 7.78 mmol h^−1^ g_cat_^−1^ [45]. The versatility in tailoring the coordination environment of MOPs underscores their research significance in electrocatalytic urea synthesis.

In electrocatalyst design, the control of metal valence states is crucial for catalyst performance, and MOPs offer the advantage of easily tunable metal centers. An emerging frontier in coordination environment engineering involves the incorporation of secondary metals to construct innovative dual‐site catalysts with complementary functionalities [46, 47, 48, 49, 50]. Unlike conventional single‐metal systems, these bimetallic architectures exploit synergistic interplay between two distinct metal centers, enabling precise tuning of reactant/intermediate adsorption energetics and molecular bond strengths. This deliberate design strategy not only optimizes intermediate adsorption–desorption kinetics but also introduces unique electronic effects: When one metal component is deliberately perturbed from its equilibrium state, the resulting lattice strain induces localized electronic redistribution. Remarkably, this strain‐mediated electronic modulation serves a dual purpose—stabilizing metal oxidation states while dramatically lowering the activation barrier for C–N coupling, as recently demonstrated in pioneering studies [51].

Significant progress has been made in the development of bimetallic catalysts for electrochemical urea synthesis, with a series of high‐performance systems successively reported, including Co–Mo [52], Cu–In [53], and Zn–Pb [54] bimetallic architectures. Among these, iron‐based bimetallic catalysts stand out due to their readily tunable electronic structures and exceptional capability for intermediate species adsorption/desorption. Notably, the introduction of Fe as a secondary metal has been demonstrated to significantly enhance catalytic performance, attributed to its unique electronic properties that facilitate key steps in the C–N coupling process. For instance, Zhang et al. engineered an isolated diatomic nanosphere catalyst with bonded Ni–Fe pairs (I‐FeNi‐DASC), achieving a record urea yield of 20.2 mmol h^−1^ g^−1^—a tenfold enhancement over monometallic Ni or Fe single‐atom catalysts [36]. Advanced characterizations, including synchrotron radiation Fourier‐transform infrared spectroscopy (SR‐FTIR) and density functional theory (DFT) calculations, revealed two thermodynamically spontaneous C–N coupling pathways: (1) *NH + *CO → *NHCO and (2) *NO → *NHO. Crucially, the bonded Fe–Ni pairs exhibited exceptionally low energy barriers of 0.21 and 0.09 eV for these steps, demonstrating their unique role in destabilizing C–N coupling transition states. Ma et al. proposed a potential‐dependent free energy variation strategy to guide the C–N coupling process [55]. The designed graphene‐supported nickel–iron diatomic catalyst (Fe–Ni–N_6_–C) exhibited remarkable performance in the electrocatalytic synthesis of urea from nitrate and carbon dioxide. The Fe–Ni diatomic pair played a synergistic role in urea production: The Fe atom with high nitrogen affinity induced the formation of monodentate N–Fe bonds with *NO_2_ and *NO intermediates. As nitrogen saturation increased, bidentate N–Fe and N–Ni bonds formed in *NHO species, thereby activating the nitrogen atom and facilitating the first C–N coupling. Both Fe and Ni sites cooperatively stabilized the reaction intermediates until the formation of *NH_2_COOH, where the nitrogen became saturated as NH_2_. Following –OH protonation, the more reactive carbon atom was attracted by the Fe site, while the unsaturated NH_2_ group remained unbound to the surface, enabling the timely occurrence of the second C–N coupling. This study has established a novel electrochemical reaction mechanism for Fe–Ni bimetallic systems in urea electrosynthesis.

In this study, we have developed a Ni–Fe bimetallic polymer (Ni‐PMDA@Fe) catalyst using a solvothermal synthesis and magnetic stirring approach for the electrocatalytic production of urea. This catalyst demonstrates superior yield and selectivity in the electrochemical conversion of NO_3_^−^ and CO_2_ to urea, surpassing the performance of the Ni‐BDC catalyst and Ni‐PMDA precatalyst. The incorporation of a tetracarboxylic acid ligand improves dispersion of active nickel sites owing to the extensive formation of Ni–O bonds, thereby enhancing catalytic activity for synergistic activation of NO_3_^−^ and CO_2_ and C–N bond formation to produce urea molecules. The incorporation of Fe establishes dual‐active‐site synergy that modulates the coordination environment and electronic state distribution of the polymer matrix. This strategic modification optimizes the adsorption configuration of C–N coupling intermediates, thereby significantly enhancing both urea yield (449.56 mg h^−1^ g^−1^) and FE (reaching 41.06%) at −0.5 V_RHE_. To further optimize the reaction condition, the influence of NO_3_^−^ concentration, CO_2_ flow rate, and the pH value of electrolyte were extensively investigated. Additionally, a techno‐economic analysis (TEA) confirms the economic viability of the Ni‐PMDA@Fe catalyzed electrochemical urea synthesis process, aligning with the principles of green chemistry. Furthermore, the strategy is anticipated to gain additional economic benefits as fossil fuel prices increase and clean energy technologies advance.

## 2 Results and Discussion

### 2.1 Catalyst Synthesis and Characterization

**SCHEME 1:**
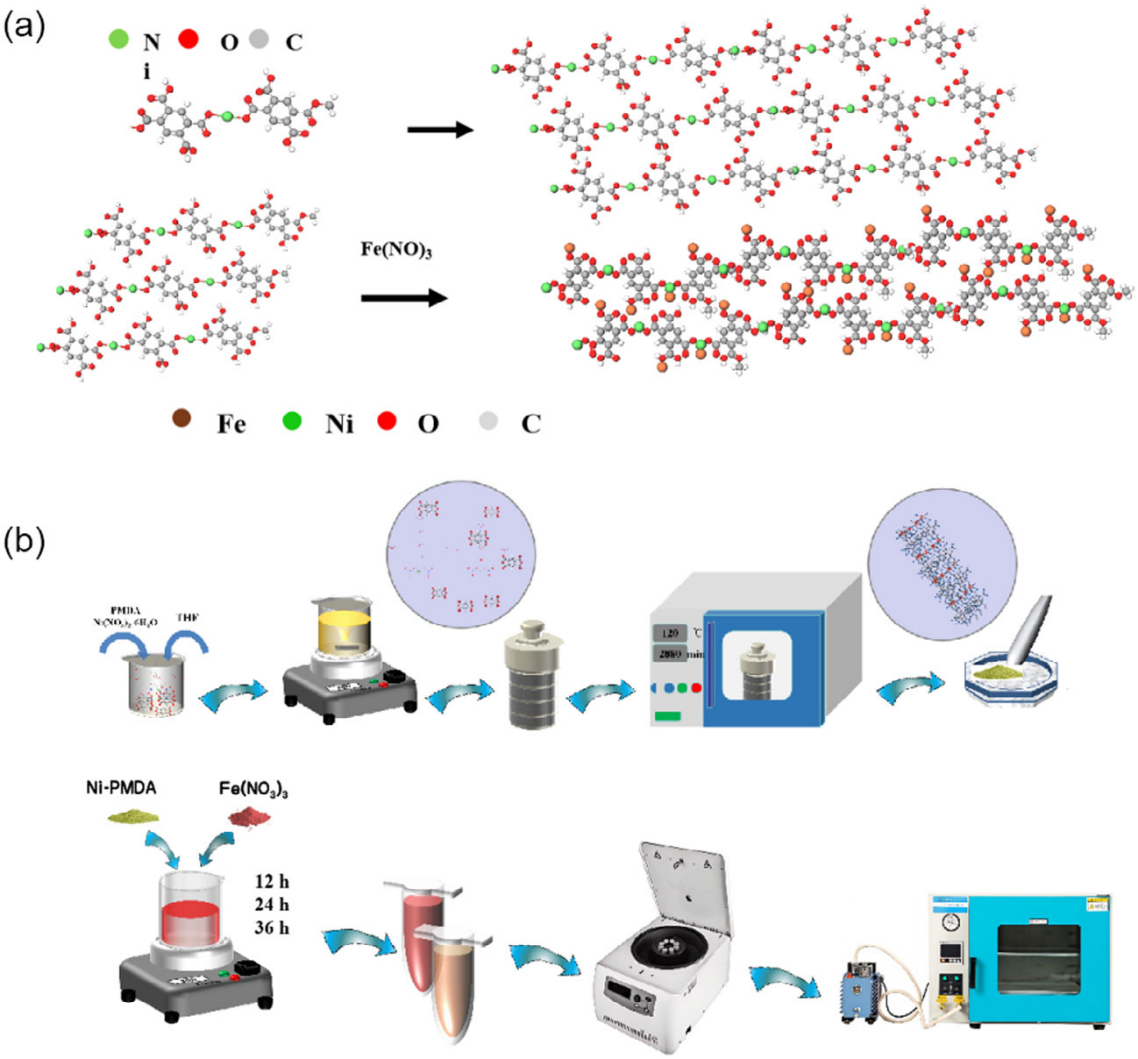
(a) Schematic illustration of Ni‐PMDA@Fe polymer synthesis. (b) Synthetic protocol for Ni–Fe bimetallic polymeric material.

Scheme 1 illustrates the synthesis protocol for the Ni‐PMDA@Fe bimetallic polymer catalyst. The Ni‐PMDA precursor was initially synthesized via a solvothermal reaction between nickel nitrate hexahydrate and pyromellitic dianhydride in tetrahydrofuran at 120°C for 48 h. Subsequently, Fe doping was accomplished through a mechanochemical approach involving magnetic stirring of the Ni‐PMDA precursor with iron(II) nitrate in anhydrous ethanol. To optimize the Fe incorporation, three samples with different stirring durations (12 , 24 , and 36 h) were prepared and designated as Ni‐PMDA@Fe‐12, Ni‐PMDA@Fe‐24, and Ni‐PMDA@Fe‐36, respectively (detailed synthesis procedures are provided in the Supporting Information). Electrochemical evaluation of urea synthesis performance identified Ni‐PMDA@Fe‐24 as the optimal catalyst, exhibiting superior activity and selectivity. ICP‐OES analysis confirmed an approximate Ni:Fe atomic ratio of 3:1 in this optimal catalyst. Interestingly, variation of Fe precursor concentration showed minimal impact on the final Fe content in Ni‐PMDA@Fe‐24, while insufficient precursor amounts led to negligible Fe incorporation. For clarity and conciseness, all subsequent references to “Ni‐PMDA@Fe” in this manuscript denote the Ni‐PMDA@Fe‐24 sample unless otherwise specified. The Ni‐BDC control catalyst was synthesized following an identical procedure with terephthalic acid substituted as the organic ligand.

**FIGURE 1:**
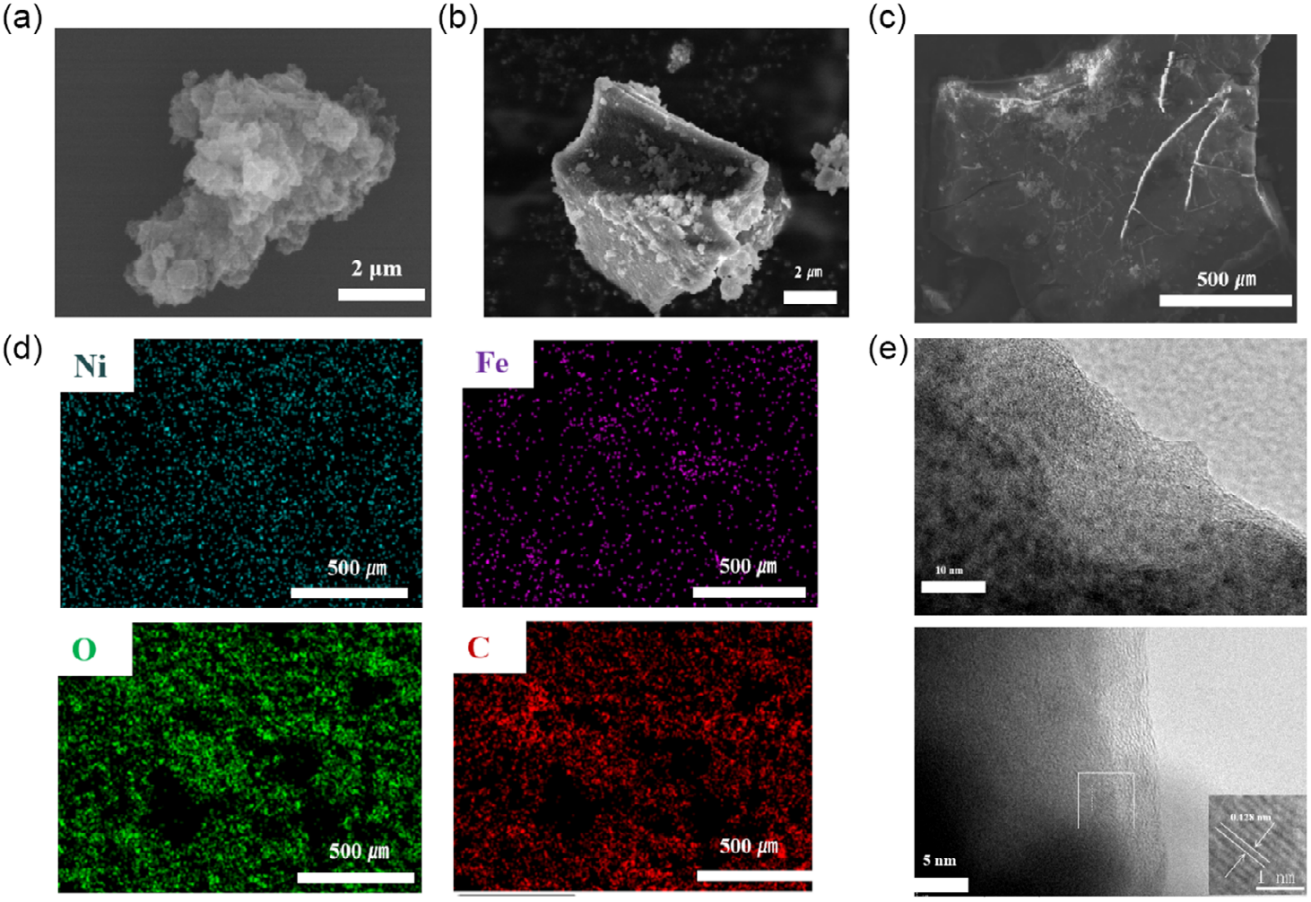
(a) SEM image of Ni‐PMDA precatalyst. (b) SEM image of Ni–Fe bimetallic polymer. (c,d) SEM image and the corresponding element mapping of Ni‐PMDA@Fe. (e) HRTEM image of Ni–Fe bimetallic polymer.

Microstructural analysis began with examination of the Ni–Fe bimetallic polymer's morphology. Figure 1b,c displays the scanning electron microscopy (SEM) images and corresponding EDS elemental mapping of the Ni‐PMDA@Fe bimetallic catalyst powder. Distinct from the Ni‐PMDA precursor's porous architecture with loose particulate aggregation (Figure 1a & Figure S1), the bimetallic polymer powder exhibits irregular block‐like morphology with no apparent porous structure, likely due to Fe incorporation filling the voids in the Ni‐PMDA matrix, resulting in densified material. Meanwhile, the as‐synthesized Ni‐BDC catalyst powder exhibits micrometer‐sized spherical particles with nanosheet structures on the surface (Figure S2), demonstrating a microstructure similar to those reported in previous studies. The EDS mapping reveals the presence of four primary elements (Fe, Ni, O, and C) throughout the catalyst structure (Figure 1d). While Fe distribution shows some regional variation at the micron scale, no large Fe aggregates are observed, suggesting that Fe species are incorporated within the polymer matrix at the sub‐micron level. This distribution pattern creates a catalyst architecture with proximal Ni and Fe sites that enables the observed synergistic effects during electrocatalysis. The high‐resolution TEM image of Ni‐PMDA@Fe reveals predominantly amorphous characteristics with limited crystalline domains (Figure 1e). Only sporadic lattice fringes with spacing of 0.128 nm are observable at particle edges, corresponding to the (2 2 0) planes of iron oxide nanoclusters formed during Fe doping. This observation aligns with our XRD analysis, confirming that the incorporation of Fe disrupts the long‐range order of the polymer framework while preserving metal‐carboxylate coordination at the molecular level.

**FIGURE 2:**
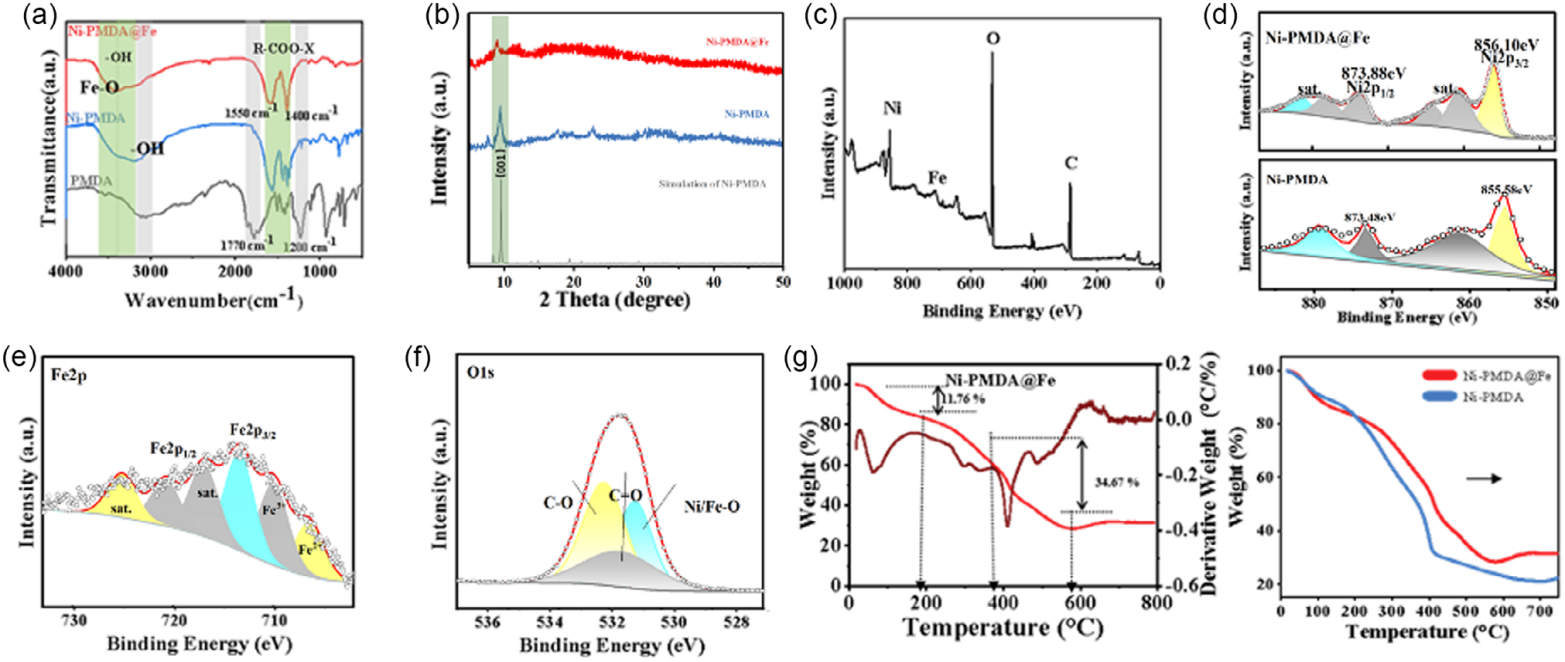
(a) FT‐IR spectra of PMDA, Ni‐PMDA precatalyst and Ni‐PMDA@Fe. (b) XRD spectra of Ni‐PMDA@Fe, Ni‐PMDA precatalyst, and simulated pattern. (c) Full‐range XPS spectrum of the Ni–Fe bimetallic coordination polymer. (d) High‐resolution Ni2p spectrum of Ni‐PMDA precatalyst and Ni‐PMDA@Fe. (e) High‐resolution Fe2p spectrum of Ni‐PMDA@Fe. (f) High‐resolution O1s spectrum of Ni‐PMDA@Fe. (g) Simultaneous thermal analysis (TGA) of Ni‐PMDA@Fe polymer and comparison with Ni‐PMDA.

Figure 2a presents the FT‐IR spectra of the synthesized Ni‐PMDA@Fe bimetallic catalyst powder. The Ni‐PMDA@Fe catalyst maintains similar functional group characteristics to the Ni‐PMDA precursor, as evidenced by the characteristic peaks at 1400 and 1550 cm^−1^, which are attributed to the RCOO‐X groups formed between the metal ions and carboxylate ligands. These peaks are distinctly different from the strong C=O absorption band at 1770 cm^−1^ and the cyclic anhydride C–O stretching vibrations between 1200–1310 cm^−1^ observed in the pure PMDA ligand. Additionally, the Ni‐PMDA@Fe spectrum exhibits not only hydroxyl functional groups but also Fe–O stretching vibrations in the 3200–3600 cm^−1^ region, suggesting coordination between the secondary metal (Fe) and oxygen atoms from carboxylate groups. According to the XRD patterns of the PMDA molecule and Ni‐PMDA (Figure S4), such metal organic polymers can be generated through synthetic route. Figure 2b presents the powder XRD patterns of Ni‐PMDA@Fe catalyst, Ni‐PMDA precursor, and its simulated patterns. The Ni‐PMDA precursor displays semicrystalline characteristics with broad diffraction features between 15°–25° and a distinct peak at 8.3°, corresponding to the (001) plane of layered metal‐carboxylate structures with monodentate coordination geometry. This pattern aligns with simulated XRD patterns of similar Ni‐based pyromellitate polymers [41]. Upon Fe incorporation, the Ni‐PMDA@Fe catalyst exhibits significantly reduced crystallinity, retaining only a weak characteristic peak at 8.5° with diminished intensity, while developing pronounced broad diffraction features in the 20°–30° range. These observations confirm that Fe doping disrupts the long‐range order of the polymer framework, resulting in a predominantly amorphous structure with short‐range metal‐carboxylate coordination. This structural transformation is consistent with the TEM observations (Figure 1e) showing indistinct lattice fringes except for sporadic crystalline domains at the edges. The FT‐IR spectrum and XRD pattern of the as‐synthesized Ni‐BDC catalyst are presented in Figures S3 and S4b, consistent with literature references [56].

To further elucidate the elemental distribution and valence states, X‐ray photoelectron spectroscopy (XPS) characterization was performed on the as‐prepared Ni‐PMDA@Fe catalyst. Figure 2c presents the XPS survey spectrum, confirming the presence of C, O, Ni, and Fe elements in the material. The higher Ni content relative to Fe suggests that Ni serves as the primary catalytic active site, while the doped Fe functions as a secondary metal center that modulates the coordination environment of the polymer. The ratio of Ni:Fe is approximately 3:1, which is aligned with the results from the characterization of ICP‐OES. Furthermore, the high‐resolution Ni 2p spectrum (Figure 2d) exhibits characteristic spin–orbit splitting. Comparative analysis between Ni‐PMDA@Fe and the Ni‐PMDA precursor (Figure S5) reveals an increased binding energy of 0.5 eV for Ni in the bimetallic polymer (from 853.7 to 854.2 eV for the main Ni 2p_3/2_ peak), providing direct evidence that Fe incorporation significantly reduces the electron density around Ni centers. This electronic modification creates more electrophilic Ni sites that preferentially adsorb electron‐rich NO_3_^−^ species, while the mixed Fe^2+^/Fe^3+^ states observed in the Fe 2p spectrum facilitate NO_3_^−^ reduction to key nitrogen intermediates (Figure 2e). This dual‐metal electronic configuration aligns with established bimetallic catalyst design principles where the d‐band center shift optimizes adsorption energetics of critical intermediates, enabling efficient C–N coupling as evidenced by our superior urea selectivity. The high‐resolution O 1s spectrum (Figure 2f) further reveals coordination bonds between Ni/Fe and oxygen, along with characteristic C–O and C=O bonds from the PMDA ligand. Comparative analysis reveals that the XPS spectra of O and C elements remain essentially unchanged before and after Fe doping (Figure S5 & Figure 2f), indicating that the incorporation of Fe exerts negligible influence on both the valence state composition and chemical configuration of these elements within the polymer matrix. Figure 2g displays the thermogravimetric analysis (TGA) profile of the Ni–Fe bimetallic organic polymer under the N_2_ atmosphere. The initial weight loss of 11.76% observed between 25°C and 120°C is attributed to the evaporation of adsorbed water and decomposition of oligomers. A significant acceleration in weight loss occurs above 300°C, indicating the onset of structural collapse in the polymeric framework. We compare the TGA curves of Ni‐PMDA@Fe and the Ni‐PMDA precursor. The Ni–Fe bimetallic polymer exhibits enhanced thermal stability compared to its monometallic counterpart, as evidenced by the higher decomposition temperature. This improvement is likely due to the modified coordination environment induced by Fe incorporation, which reinforces the structural integrity of the polymer matrix.

The comprehensive material characterization demonstrates that the Ni‐PMDA@Fe catalyst successfully incorporates Fe as a secondary metal while maintaining the fundamental metal–organic polymeric chain structure, thereby constructing a stable Fe–Ni bimetallic organic polymer material. The observed micron‐scale heterogeneity in Fe distribution does not compromise catalytic performance, as the intimate contact between Fe and Ni species at the nanoscale, evidenced by XPS analysis showing electronic interaction between these metals, enables the synergistic effects critical for C–N coupling. Notably, the introduction of Fe modifies the coordination environment of the polymer and alters the electron density distribution around Ni centers, thereby enhancing the catalytic activity toward electron‐rich NO_3_^−^ species. Through these systematic characterizations, we have established a clear understanding of the valence state composition, crystalline characteristics, and morphological features of the Ni‐PMDA@Fe bimetallic polymer. These fundamental insights provide a solid foundation for subsequent electrocatalytic performance evaluation.

### 2.2 Electrochemical Urea Synthesis

**FIGURE 3:**
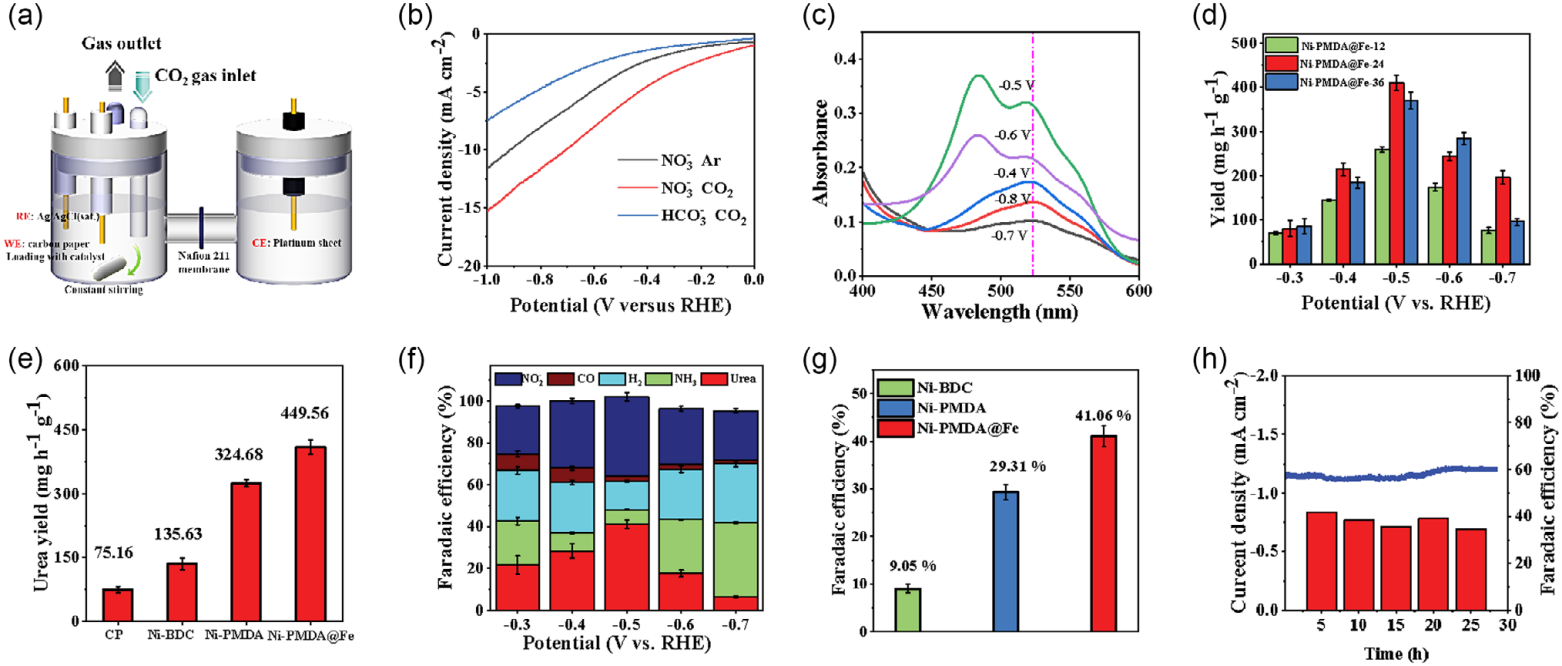
(a) Architecture of H‐cell electrochemical configuration employing three‐electrode setup. (b) The polarization curves of Ni‐PMDA@Fe under different catalytic conditions. (c) Urea adsorption profile of Ni–Fe bimetallic polymer catalyst. (d) Average urea yield of Ni‐PMDA@Fe at different electrolysis potentials. (e) Catalyst–performance correlation in electrochemical urea synthesis. (f) Comparative Faradaic efficiencies for urea and competing reduction products. (g) Comparative analysis of urea selectivity in Ni‐BDC, Ni‐PMDA, and Ni‐PMDA@Fe catalysts. (h) Catalyst stability testing.

Figure 3a and Figure S6 illustrate the H‐type electrolytic cell and three‐electrode system employed in this study. The electrochemical performance of the catalysts was evaluated using a CHI 760E electrochemical workstation coupled with an air‐tight H‐type electrolytic cell. Throughout the experiments, the system's hermetic integrity was rigorously maintained. The resulting catalyst powder was then mixed with acetylene black, isopropanol, and Nafion solution to prepare the ink mixture, which was applied to conductive carbon cloth using a pipette. The electrocatalytic performance was evaluated using a conventional three‐electrode system, with the catalyst‐coated carbon cloth as the working electrode, an Ag/AgCl (3 M KCl) reference electrode, and a platinum wire counter electrode. The working electrode was fabricated by depositing catalyst ink onto a carbon cloth substrate. All measured potentials were converted to the reversible hydrogen electrode (RHE) scale using the Nernst equation for standardized potential reporting. The electrochemical measurements were performed in KNO_3_ electrolyte as the NO_3_^−^ source, with high‐purity CO_2_ gas continuously purged at a controlled flow rate using a mass flow controller. All experiments were conducted at 25°C under ambient conditions. Prior to testing, the carbon cloth‐supported catalyst was electrochemically activated through cyclic voltammetry (CV) scanning between 0 and 1.0 V_RHE_ for 200 cycles. Subsequently, linear sweep voltammetry (LSV) was performed to determine the optimal potential window and evaluate the catalytic response toward NO_3_^−^ reduction. The target product, urea, was quantified using the diacetyl monoxime method (Figure S6), Ammonia concentration was determined by the indophenol blue method (Figure S7), and nitrite concentration was measured via *N*‐(1‐naphthyl)‐ethylenediamine dihydrochloride spectrophotometry (Figure S8). The main gaseous byproducts were analyzed using an SP‐3400 gas chromatograph. Figure 3b shows the LSV curves measured for the Ni‐PMDA@Fe catalyst. A comparison reveals that the LSV curve obtained in the presence of both KNO_3_ and CO_2_ exhibits the highest current density, significantly surpassing those measured with only CO_2_ or KNO_3_. This serves as strong evidence of the catalyst's response to both CO_2_ and NO_3_^−^ substrates. Based on the LSV curves, we set the reaction potential window to −0.3 to −0.7 V_RHE_. We systematically optimized the electrolytic reaction conditions. Figure S9 presents the urea production yield and FE measured under controlled variations of nitrate concentration, CO_2_ partial pressure, and electrolyte pH, respectively. Comprehensive parametric studies revealed that the catalyst exhibited optimal performance when employing a CO_2_ flow rate of 30 mL min^−1^, a KNO_3_ concentration of 0.05 mol L^−1^, and either acidic or neutral electrolyte conditions. Based on these findings, we employed a mixed electrolyte system comprising 35 mL of 0.05 M KNO_3_ and 35 mL of 0.05 M KHCO_3_ (total volume 70 mL), while maintaining the CO_2_ flow rate at 30 mL min^−1^ throughout the electrochemical measurements.

**FIGURE 4:**
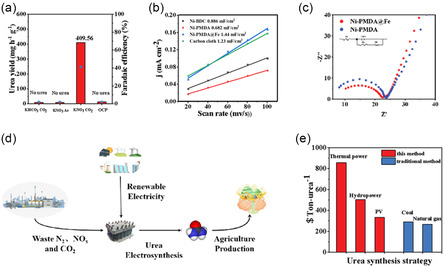
(a) Urea yield measured under different reaction substrates. (b) ECSA comparison of various catalysts. (c) EIS comparison of Ni‐PMDA@Fe and Ni‐PMDA precatalyst. (d) Schematic diagram of electrochemical synthesis of urea and (e) TEA of Ni‐PMDA with traditional industrial methods.

Figure 3c presents the UV–vis absorption spectra of the Ni‐PMDA@Fe catalyst at various applied potentials, exhibiting a characteristic absorption peak at 525 nm. Based on this observation, we established a linear correlation between absorbance and urea concentration. Notably, the highest absorption intensity was achieved at −0.5 V_RHE_, indicating optimal catalytic activity at this potential (Figure S10). The incorporation of Fe species was found to be strongly dependent on the stirring duration during catalyst synthesis. To systematically investigate this relationship, we compared the urea production yields of Ni‐PMDA@Fe catalysts prepared with different magnetic stirring times (12, 24, and 36 h), as shown in Figure 3d. The Ni‐PMDA@Fe‐12 catalyst demonstrated negligible urea yield enhancement, which we attribute to insufficient Fe incorporation and coordination due to the relatively short stirring period. In contrast, the Ni‐PMDA@Fe‐24 catalyst exhibited significantly enhanced urea production, outperforming both Ni‐PMDA and Ni‐BDC monometallic catalysts (Figure 3e). This improvement can be ascribed to the optimal formation of Ni–Fe dual‐metal active sites. Interestingly, extending the stirring time to 36 h (Ni‐PMDA@Fe‐36) did not further improve catalytic performance, indicating that the active site formation reaches completion within 24 h. The Faradaic efficiencies (FEs) of various reaction products are presented in Figure 3f. The main product urea achieved its maximum FE of 41.06% at −0.5 V_RHE_, demonstrating significant superiority over both Ni‐BDC and Ni‐PMDA catalysts (Figure 3g & Figure S11). This enhancement can be attributed to the synergistic electronic interaction between Ni and Fe sites that optimizes the reaction pathway for C–N coupling. Notably, LSV measurements reveal significantly enhanced current density only when both NO_3_^−^ and CO_2_ are present (Figure 3b), providing direct evidence of cooperative substrate activation. Additionally, the Ni‐PMDA@Fe catalyst exhibited superior catalytic performance in co‐reduction of NO_3_^−^ and CO_2_ compared to recent advances, presenting considerable FE and urea yield at relatively lower potential (Table S1). Furthermore, the product distribution further supports this mechanism: urea formation occurs exclusively when both substrates are available (Figure 4a), while competing ammonia production is effectively suppressed. This behavior is characteristic of well‐designed bimetallic systems where Fe sites preferentially adsorb and reduce NO_3_^−^ to *NH_*x*_ intermediates while electron‐deficient Ni sites activate CO_2_, with the optimal spatial arrangement enabling efficient C–N bond formation. The stability test confirms the robustness of this dual‐site architecture, maintaining >30% FE over 30 h of continuous operation at −0.5 V_RHE_ (Figure 3h). The catalyst maintained a stable current density of −1.2 mA cm^−2^ throughout the test. Poststability characterization revealed that the urea FE consistently remained above 30% in periodic sampling tests, confirming the excellent catalytic durability of our material.

To elucidate the carbon and nitrogen sources in the urea product, we systematically controlled the reaction inputs and monitored urea formation. Under standard electrolytic conditions (35 mL of 0.05 M KNO_3_ + 35 mL of 0.05 M KHCO_3_ with 30 mL min^−1^ CO_2_ flow), urea production was clearly detected. However, when either CO_2_ or nitrate ions were excluded from the reaction system, no urea formation was observed in the products. These control experiments conclusively demonstrate that the carbon in urea originates exclusively from CO_2_ rather than carbonate species, and the nitrogen derives specifically from nitrate ions rather than atmospheric nitrogen compounds or other potential contaminants (Figure 4a). The catalytic kinetics of various catalysts were systematically investigated. Figure 4b presents the double‐layer capacitance (*C*_dl_) determined by CV measurements (Figure S12), which serves as an indicator of electrochemical active surface area (ECSA). The linear relationship between scan rates and non‐Faradaic capacitive currents in the potential window yields slopes corresponding to *C*_dl_ values. Notably, the Ni‐PMDA@Fe catalyst exhibits a significantly higher *C*_dl_ compared to both Ni‐PMDA and Ni‐BDC, indicating a greater ECSA that confers substantial kinetic advantages. Complementary electrochemical impedance spectroscopy (EIS) measurements were performed to compare charge transfer characteristics, with the corresponding Nyquist plots shown in Figure 4c. The semicircle radius in the high‐frequency region, which reflects charge transfer resistance, demonstrates that the Ni–Fe bimetallic catalyst possesses markedly lower impedance than the Ni‐PMDA precursors. Moreover, the structural stability and catalytic durability of Ni‐PMDA@Fe catalyst was verified by the SEM image (Figure S13) and XRD pattern (Figure S14) after long‐term electrolysis.

The exceptional urea selectivity of Ni‐PMDA@Fe can be rationalized through a dual‐site reaction mechanism supported by our experimental observations and recent literature. Unlike single‐metal catalysts where competing reaction pathways lead to undesired byproducts (primarily NH_3_ and HCOOH), the Ni–Fe configuration establishes a synergistic catalytic cycle for C–N coupling. Based on our control experiments (Figure 4a) and product distribution analysis, we propose the following mechanism: (1) Fe sites with their higher affinity for nitrogen‐containing species preferentially adsorb and reduce NO_3_^−^ to *NO and subsequently to *NH_2_ intermediates; (2) adjacent electron‐deficient Ni sites (evidenced by XPS binding energy shifts) activate CO_2_ to form *COOH/*CO intermediates; (3) the optimal spatial arrangement of Ni and Fe sites in the polymer matrix (confirmed by EDS mapping) enables proximity‐driven C–N coupling between *NH_2_ and *CO species to form the *NHCO intermediate; (and 4) further hydrogenation and coupling with another *NH_2_ yields urea. This mechanism explains why our catalyst achieves significantly higher urea selectivity (41.06% FE) than monometallic controls—only the precisely tuned electronic and geometric configuration of the dual‐metal sites provides the appropriate binding energy window for stabilizing the critical *NHCO intermediate while preventing over‐hydrogenation to NH_3_. Recent computational studies on similar Ni–Fe systems have identified exceptionally low energy barriers (0.09–0.21 eV) for these C–N coupling steps when compared to monometallic counterparts, supporting our experimental observations.

### 2.3 TEA

According to statistics, global fertilizer demand reached 204 million tons in 2023, an increase of about 8.5% compared to prepandemic levels. Urea, which accounts for 85% of the total nitrogen fertilizer production, is traditionally manufactured through the energy‐intensive Haber–Bosch process. Conventional urea production relies heavily on coal and natural gas, representing a major source of CO_2_ emission [57]. Calculations show that producing one kilogram of urea consumes 22–27 megajoules of energy and emits 3.5–4.1 kg of CO_2_ [58, 59]. In contrast, our electrocatalytic approach offers significant environmental advantages. Based on life cycle assessment methodology, the carbon footprint of our electrochemical process varies with electricity sources: When powered by coal electricity ($0.09/kWh), the process emits approximately 2.8 kg CO_2_ per kg of urea; this value decreases to 1.2 kg CO_2_ with hydropower ($0.043/kWh) and further reduces to 0.7 kg CO_2_ with solar power ($0.025/kWh). Moreover, this technology provides additional environmental benefits by simultaneously treating wastewater nitrate pollutants and fixing CO_2_, eliminating approximately 1.2 kg of nitrogen pollutants and 0.6 kg of CO_2_ emissions per kg of urea produced. In total, when powered by renewable energy, our electrochemical process can reduce carbon emissions by more than 80% compared to the conventional Haber–Bosch route.

Given the high energy consumption and emissions of traditional processes alongside the excellent electrocatalytic performance of our catalyst (Figure 4d), we conduct a brief economic evaluation of the catalyst's commercial application using the TEA model (Table S2) [60, 61]. According to the widely accepted definition, TEA = LCC + OPEX, where LCC represents “Levelized Capital Cost,” primarily referring to the costs of catalyst materials, preparation, and reaction equipment. In this method, the catalyst is made from commonly used Ni nitrate and anhydride, and synthesized using a low‐cost, energy‐efficient solvothermal method at only 120°C, making these costs negligible. OPEX represents “Operating Expenses,” mainly referring to the energy consumption of the electrochemical reaction. Since the electrochemical reaction is carried out at room temperature and atmospheric pressure with a neutral or weakly acidic electrolyte, only the electrical energy consumed needs to be considered, giving this electrochemical synthesis a significant advantage over traditional methods. In this experiment, each gram of Ni‐PMDA@Fe catalyst produces 449.56 mg of urea per hour with a FE of about 41.06%, a stable current density of 3.12 mA cm^−2^, using a standard 100 L reactor and approximately 3.36 m^2^ of total electrode area. With an electricity cost of $0.09/kWh for conventional industrial coal power, we analyze the TEA model. Detailed assumptions and calculation results for LCC and OPEX are shown in Table S2. The calculation shows that the TEA for this electrochemical synthesis is approximately $51.44 kmol^−1^_urea_. We also note that the primary expense in TEA is the cost of electricity. By using cheaper electricity sources such as wind power ($0.072/kWh), hydropower ($0.043/kWh), or solar power ($0.025/kWh), operational costs can be further reduced. Figure 4e compares the costs of this method with traditional urea production. The cost for this method is $856/ton, which can be reduced to $504 and $332/ton with hydropower or solar power, respectively. These costs are approaching those of traditional coal‐based and natural gas‐based urea production and this method becomes more advantageous as fossil fuel prices rise [62]. Importantly, the electrochemical synthesis method, as a green synthesis strategy, overcomes the reliance on traditional ammonia synthesis methods, reducing greenhouse gas emissions and fossil fuel consumption while decreasing the carbon footprint for a more sustainable economy.

## 3 Conclusion

To address the environmental challenges posed by nitrate‐laden industrial wastewater and greenhouse gases, as well as the high emissions and energy consumption associated with conventional urea production, this study developed a cost‐effective and highly efficient Ni–Fe bimetallic–pyromellitic acid polymer catalyst (Ni‐PMDA@Fe) using transition metal catalysts and common anhydride ligands. The synthesized metal–organic polymer exhibits structural stability, highly dispersed active sites, and tunable coordination environments, demonstrating significant advantages as a catalyst for urea electrosynthesis. Experimental results revealed a maximum urea yield of 449.56 mg h^−1^ g^−1^ and a peak urea selectivity of 41.06%. The incorporation of Fe was found to modulate the coordination environment, increase the number of active sites, and enhance charge transfer efficiency, thereby facilitating the catalytic process of C–N coupling reactions. Finally, a TEA was conducted to evaluate the operational costs and application prospects of the electrocatalytic urea synthesis approach, demonstrating its potential for sustainable and economical urea production.

## Supporting Information

Additional supporting information can be found online in the Supporting Information section. The authors have cited additional references within the Supporting Information. ^[S1‐S19]^ **Supporting Fig. S1:** The scanning electron microscopy (SEM) images and corresponding EDS elemental mapping of Ni‐PMDA precursor. **Supporting Fig. S2:** The scanning electron microscopy (SEM) images of the Ni‐BDC metal‐organic polymer. **Supporting Fig. S3:** FTIR spectrum of Ni‐BDC. **Supporting** **Fig. S4:** XRD patterns of Ni‐PMDA,Ni‐BDC, and PMDA Raw Materials. **Supporting** **Fig. S5:** (a) XPS survey spectra of Ni‐PMDA. (b) high‐resolution Ni2p_3/2_ spectrum of Ni‐PMDA. (c) high‐resolution O1s spectrum of Ni‐PMDA. (d) high‐resolution C1s spectrum of Ni‐PMDA. **Supporting** **Fig. S6:** the experimental setup of this work. **Supporting Fig. S6:** (a) UV‐vis absorbance value at 525 nm for urea and (b) standard absorption curve. **Supporting** **Fig. S7:** (a) UV‐vis absorbance value at 525 nm for NH_3_ and (b) standard absorption curve. **Supporting Fig. S8:** (a) UV‐vis absorbance value at 525 nm for NO_2_^−^ and (b) standard absorption curve. **Supporting Fig. S9:** the urea production yield and Faradaic efficiency measured under different the electrolytic reaction conditions. **Supporting** **Fig. S10:** (a) Current density profiles of Ni‐PMDA@Fe at various applied potentials. (b) Urea yield rates of Ni‐PMDA@Fe under different electrocatalytic potentials. **Supporting Fig. S11:** (a) Current density curves and (b) urea yield of Ni‐BDC catalysts at different potentials; (c) average urea and NH_3_ yield of Ni‐BDC. **Supporting Fig. S12:** (a) cylic voltammetry curves of Ni‐PMDA@Fe at different scan rates. (b) cylic voltammetry curves of Ni‐PMDA at different scan rates. (c) cylic voltammetry curves of Ni‐BDC at different scan rates. **Supporting Fig. S13:** SEM image of Ni‐PMDA@Fe after electrolysis. **Supporting** **Fig. S14:** XRD patterns of Ni‐PMDA@Fe after electrolysis. **Supporting** **Table S1:** Recent advances in electrochemical synthesis of urea (H‐cell). **Supporting** **Table S2:** The detailed process of technical and economic assessment.

## Author Contributions

**Daming Feng:** formal analysis; methodology; writing – original draft; funding acquisition, **Zhenghao Lyu:** investigation; writing – original draft, **Qian Zhang:** investigation; formal analysis, **Hui Li:** visualization, **Fengxia Wei:** validation; write – review & editing, **Zhenglong Li:** validation; methodology, **Hongge Pan:** validation, **Tianyi Ma:** supervision; funding acquisition.

## Funding

This study was supported by Fundamental Research Funds for Public Universities in Liaoning (LJ212510140013), Liaoning Provincial Science and Technology Program Joint Project (2025‐MSLH‐293), Australian Research Council (FT210100298, DP220100603, LP210200504, LP220100088, LP230200897, IH240100009, CRCPXIII000077).

## Conflicts of Interest

The authors declare no conflicts of interest.

## Supporting information

Supplementary Material

## Data Availability

The data that support the findings of this study are available in the supplementary material of this article.
