# Supplementary material for: Ni–Fe Dual‐Site Polymer Catalyst for High Performance and Stable Electrochemical Urea Synthesis from CO2 and NO3 −
Source: ChemSusChem. 2026 Mar 12;19(6):e202502676. doi: 10.1002/cssc.202502676 (PMC12981951; doi:10.1002/cssc.202502676)
Supplement: Supplementary file 1 — Supplementary Material [file CSSC-19-e202502676-s001.pdf]

Supporting Information

Ni-Fe Dual-Site Polymer Catalyst for High Performance and Stable Electrochemical Urea Synthesis from CO_2 and NO_3^-

Daming Feng[a,b], Zhenghao Lyu[a], Qian Zhang[a], Hui Li[c,d], Fengxia Wei[e], Zhenglong Li[f], Hongge Pan[f], Tianyi Ma[c,d]

[a] College of Chemistry, Liaoning University, Shenyang China, 110036.

[b] State Key Laboratory of Biobased Transportation Fuel Technology，Zhejiang University，Hangzhou, 310058, China

[c] Centre for Atomaterials and Nanomanufacturing (CAN), School of Science, RMIT University, Melbourne, VIC, 3000, Australia

[d] ARC Industrial Transformation Research Hub for Intelligent Energy Efficiency in Future Protected Cropping (E2Crop) Melbourne, VIC, 3000, Australia

[e] Mark Wainwright Analytical Center, University of New South Wales, Sydney, New South Wales 2052, Australia

[f] Institute of Science and Technology for New Energy，Xi’an Technological University, Xi’an 710021

E-mail: tianyi.ma@rmit.edu.au.

Materials

Nickel nitrate hexahydrate($Ni(NO_3)_2·6H_2O$), diacetylmonoxime ($C_4H_7NO_2$, AR), thiosemicarbazide (CH_5N_3S, 99 %) , sodium salicylate ($C_7H_6O_3Na$) and sodium nitroferricyanide dihydrate ($C_5FeN_6Na_2O·2H_2O$) were purchased from Aladdin Ltd.(Shanghai, China). Potassium nitrate (KNO_3) and Sulfonamide ($C_6H_8N_2O_2S$, AR) were purchased from China National Medicines Co., Ltd. Pyromellitic dianhydride (PMDA, $C_{10}H_2O_6$) and *N*-(1-aphthalene)-ethylenediamine hydrochloride ($C_{12}H_{14}N_2·2HCl$) were purchased from MACKLIN Ltd. (Shandong, China). Tetrahydrofuran (THF)were purchased from Tianjing Fuyu Fine Chemical Co., Ltd. Sulfuric acid (H_2SO_4, 98 %), phosphoric acid (H_3PO_4) and ethanol (C_2H_5OH) were purchased from Tianjin Yongda Chemical Reagent Co., Ltd. Sodium hypochlorite solution (NaClO) was purchased from Sinopharm Chemical Reagent Co., China. Iron chloride($FeCl_3$, 99.9 %), 1,4-dicarboxybenzene(PTA), Potassium hydroxide (KOH), potassium bicarbonate($KHCO_3$), Potassium sodium tartrate tetrahydrate ($C_4H_4O_6KNa·4H_2O$) , Potassium hydroxide (KOH) and ammonium chloride (NH_4Cl) were purchased from Tianjin Damao Chemical Reagent Co., Ltd. Nafion (5 wt%) and Nafion 117 membrance were purchased from Shanghai Hesen Electric Co., Ltd. CO_2 gas(≥ 99.999 %), Ar (≥99.999 %) gas were provided by Shenyang Zhaote Gas Co., LTD. Ultrapure water used throughout the whole experiment was purified through a Millipore system. All chemicals used were of analytical reagent grade and used without further purification.

Preparation of Ni-PMDA

1.6 mmol $Ni(NO_3)_2 · 6H_2O$ was first dissolved in 30 ml tetrahydrofuran (THF), 0.6 mmol pyromellitic dianhydride (PMDA), were dispersed in 20 ml THF, then the PMDA were slowly dripped into the metal salt solution. The mixed were solution ultrasonically oscillated for 30 min to obtain a homogeneous solution and transferred to 100 ml hydrothermal reaction kettle at 120 ℃ for 48 h. The resulting product was filtered by sand core filtration device and dried in the oven at 60 ℃ for 24 h.

Preparation of Ni-BDC

The preparation procedure of Ni-BDC was similar to that of Ni-PMDA, except that PMDA was replaced by PTA.

Preparation of Ni-PMDA@Fe

The doping of Fe metal was performed through a mechanochemical strategy employing magnetic stirring. The specific procedure was as follows: First, 50 mg of the Ni-PMDA polymer precursor and 50 mg of $Fe(NO_3)_2 \cdot 9H_2O$ solid were accurately weighed and separately dissolved in 25 mL of anhydrous ethanol. The mixtures were ultrasonicated for 30 min at room temperature to ensure uniform dispersion. Subsequently, the two solutions were combined and transferred to a magnetic stirrer, where continuous stirring facilitated the insertion of Fe metal into the Ni-PMDA polymer framework.

After stirring, the resulting Ni-Fe polymer was transferred to a centrifuge tube and subjected to centrifugation to isolate the solid particles. The collected solids were then washed with anhydrous ethanol and centrifuged repeatedly to remove impurities. This washing-centrifugation cycle was performed three times to ensure the purity of the final polymer. The purified solid was dried to obtain the Ni-PMDA@Fe polymer. To control the Fe doping concentration, different magnetic stirring durations (12 h, 24 h, and 36 h) were applied, yielding three distinct samples labeled as Ni-PMDA@Fe-12, Ni-PMDA@Fe-24, and Ni-PMDA@Fe-36, respectively.

Preparation of the working electrode

Typically, 3 mg of catalyst, 3 mg Acetylene black and 30 μL of Nafion solution (5 wt%) were dispersed in 170 μL ethanol by means of sonication for 1 h to form a homogeneous black ink. The cathode was prepared by loading the ink onto a carbon cloth electrode (1 cm × 2 cm, the practically immersing area in the electrolyte was 1

cm × 1 cm).

Characterization

The UV-vis spectra were obtained on a Shimadzu UV-2600 spectrophotometer. The composition of functional groups and chemical bonds in the catalyst material was determined by the Nicolet IS5 Fourier transform infrared spectroscopy (FT-IR). The X-ray diffraction (XRD) patterns were obtained on a Bruker (Germany) D8 Advance diffractometer with Cu Kα radiation in the range of 5°-60° (2θ). The scanning electron microscope (SEM) images and EDX mappings were acquired using a Hitachi SU-8010 equipped with an EDX analyzer operated at an accelerating voltage of 15 kV. The transmission electron microscopy (TEM) morphology of all samples were obtained on a JEM-2100 with 200 kV test voltage. X-ray photoelectron spectroscopy (XPS) measurement was carried out on an ESCALAB Mk II (Vacuum generators) spectrometer with an Al K X-ray source (240 W).

Electrochemical measurements

All electrochemical characterizations were performed using a CHI 760E (Shanghai, Chenhua Co., China) workstation coupled with a three-electrode system in a a typical H-cell cell separated by Nafion117 membrane. The Nafion membrane was heated in H_2O_2 (3 %) aqueous solution at 80 ℃ for 1 h, then heated in H_2SO_4 (0.5 M) aqueous solution at 80 ℃ for 1 h, finally treated in H_2O aqueous solution at 80 ℃ for another 1 h in sequence. Typically, a carbon cloth was used as the working electrode, an Ag/AgCl (in saturated KCl electrolyte) electrode was used as the reference electrode and a platinum foil electrode as the counter electrode. Potential without iR-compensated were converted to RHE scale via the following equation: $E_{RHE} = E_{Ag/AgCl} + 0.059 \times pH + 0.205$ (pH = 8.2). All experiments were carried out at room temperature (25 ^oC).

Determination of urea concentration

The urea concentration was determined by the diacetyl monoxime method. In general, 5 g diacetyl monooxime (DAMO) and 100 mg thiosemicarbazide (TSC) were dissolved in distilled water and diluted to 1000 mL, denoted as DAMO-TSC solution. Then 100 ml concentrated phosphoric acid (H_3PO_4), 300 ml concentrated sulfuric acid (H_2SO_4) were dissolved in 600 ml distilled water, then 100 mg $FeCl_3$ was dissolved in the above solution, denoted as the acid-ferric solution. When tested, 2 ml sample solution, 2 ml DAMO-TSC solution and 4 mL acid-ferric solution were mixed. Next, the mixed solution was heated to 100 ℃ and maintained for 15 min. When the solution cooled to 25 ℃, the UV-Vis absorption spectrum was collected at a wavelength of 525 nm.The standard curve was calibrated using the standard urea solution, after three consecutive independent tests, the measured absorbance and urea concentration showed a good linear relationship.

FE was calculated according to the following equation:

$FE_{urea} = 16 \times F \times C_{urea} \times V / (60.06 \times Q)$

The rate of formation was calculated using the following equation:

urea yield rate = $C_{urea} \times V / (m_{cat} \times t)$

Where F is Faraday constant (96500 C mol^{-1}), C_{urea} is the concentration of urea; V is the volume of the cathodic reaction electrolyte; Q is the quantity of electricity; t is the time of process; m_{cat} is the mass of catalyst loaded at the carbon cloth.

Determination of NH_3 concentration

The NH_3 concentration was determined by the indophenol blue method. In general, 5 g sodium salicylate ($C_7H_6O_3Na$) and 5 g Potassium sodium tartrate tetrahydrate ($C_4H_4O_6KNa \cdot 4H_2O$) were dissolved in 100 ml 0.1 M KOH solution as the color developing agent, 0.05 M NaClO was used as an oxidant, and sodium nitroferricyanide (1 wt%) was used as the catalyst. When tested, 4 ml sample solution, 4 ml color developing agent and 2 ml oxidant and 0.4 ml catalyst were mixed and standing at room temperature for 60 min. the UV-Vis absorption spectrum was

collected at a wavelength of 655 nm. The standard curve was calibrated using the NH_4Cl concentration solution, after three consecutive independent tests, the measured absorbance and NH_4Cl concentration showed a good linear relationship.

FE was calculated according to the following equation:

$FE_{NH3} = 8\ F \times C_{NH3} \times V / (17 \times Q)$

The rate of formation of NH3 was calculated using the following equation:

NH_3 yield rate = $C_{NH3} \times V / (m_{cat} \times t)$

Where F is Faraday constant (96500 C mol^{-1}), C_{NH3} is the concentration of NH_3; V is the volume of the cathodic reaction electrolyte; Q is the quantity of electricity; t is the time of process; m_{cat} is the mass of catalyst loaded at the carbon cloth.

Determination of NO_2^- concentration

The NO_2^- concentration was determined by *N*-(1-aphthalene)-ethylenediamine hydrochloride spectrophotometry method. In general, 0.1g *N*-(1-aphthalene)-ethylenediamine hydrochloride, 1.0 g sulfonamide and 2.94 ml concentrated phosphoric acid(H_3PO_4) was dissolved in 50 ml ultrapure water as Criess reagent. When tested, 2 ml sample solution, 2 ml Criess reagent and 4 ml ultrapure water were mixed and standing at room temperature for 15 min. The UV-Vis absorption spectrum was collected at a wavelength of 540 nm. The standard curve was calibrated using the $NaNO_2$ concentration solution. After three consecutive independent tests, the measured absorbance and $NaNO_2$ concentration showed a good linear relationship.

FE of NO_2^- was calculated according to the following equation:

$FE_{NO2} = 2\ F \times C_{NO2^-} \times V / (46 \times Q)$

Where F is Faraday constant (96500 C mol^{-1}), C_{NO2^-} is the concentration of NO_2^-; V is the volume of the cathodic reaction electrolyte; Q is the quantity of electricity.

Determination of CO and H_2

The CO and H_2 was determined by the SP3400 Gas chromatograph with FID and TCD detectors.

FE of CO was calculated according to the following equation:

$FE_{CO} = 2\ F \times C_{CO} \times V / (28 \times Q)$

FE of H_2 was calculated according to the following equation:

$FE_{H2} = 2\ F \times C_{H2} \times V / (2 \times Q)$

Where F is Faraday constant (96500 C mol^{-1}), C_{CO} is the concentration of CO, C_{H2} is the concentration of H_2;V is the volume of gas collected per unit time; Q is the quantity of electricity.

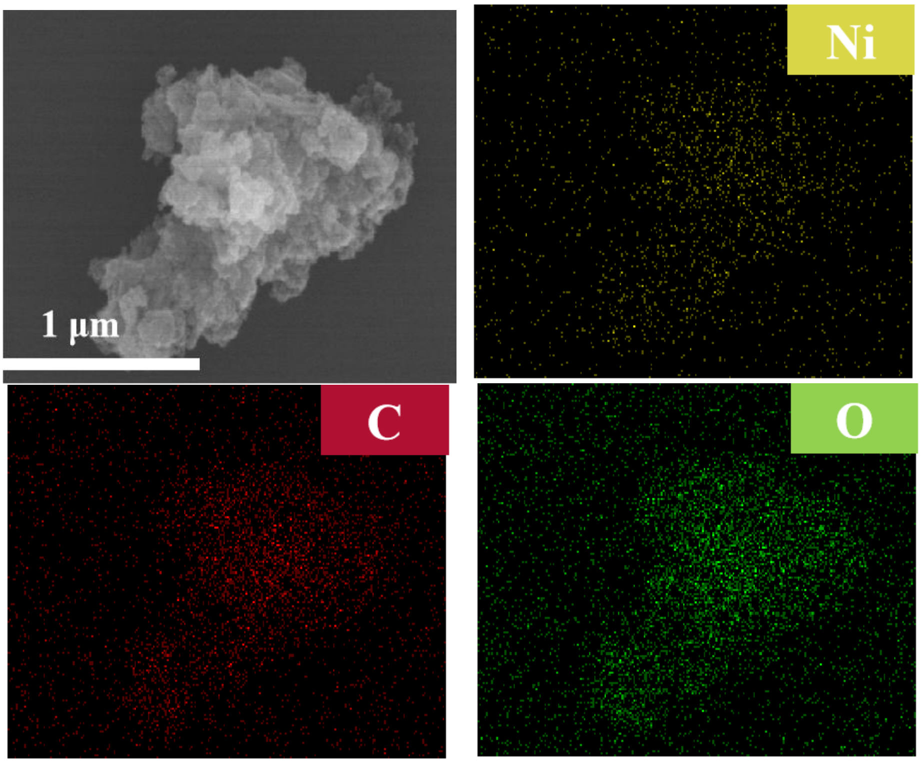

Figure S1 the scanning electron microscopy (SEM) images and corresponding EDS elemental mapping of Ni-PMDA precursor.

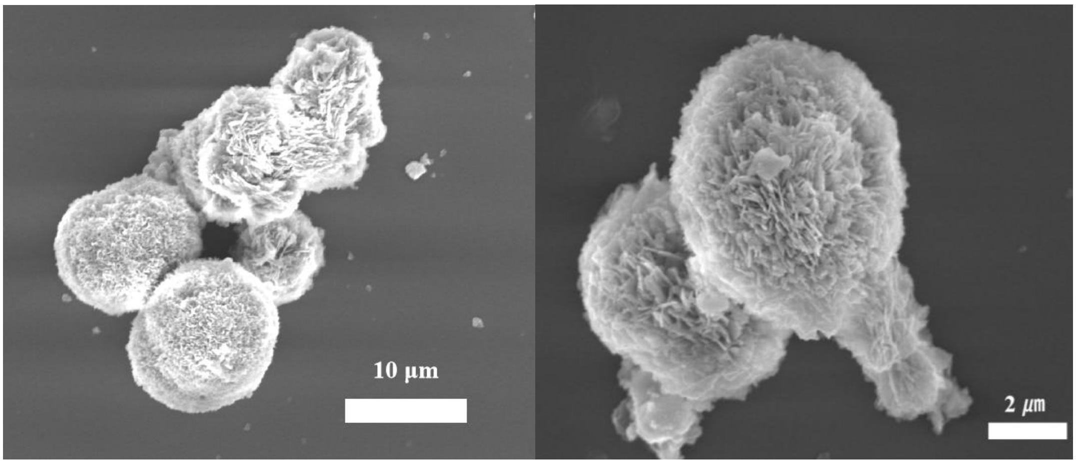

Figure S2 The scanning electron microscopy (SEM) images of the Ni-BDC metal-organic polymer.

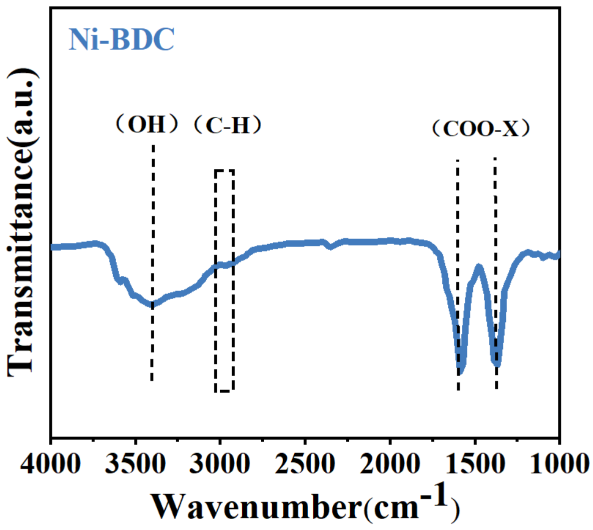

Figure S3 FTIR spectrum of Ni-BDC.

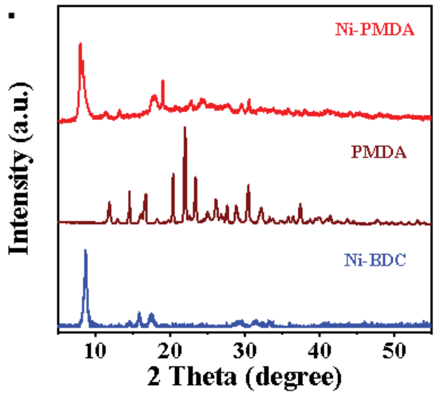

Figure S4 XRD patterns of Ni-PMDA,Ni-BDC, and PMDA Raw Materials.

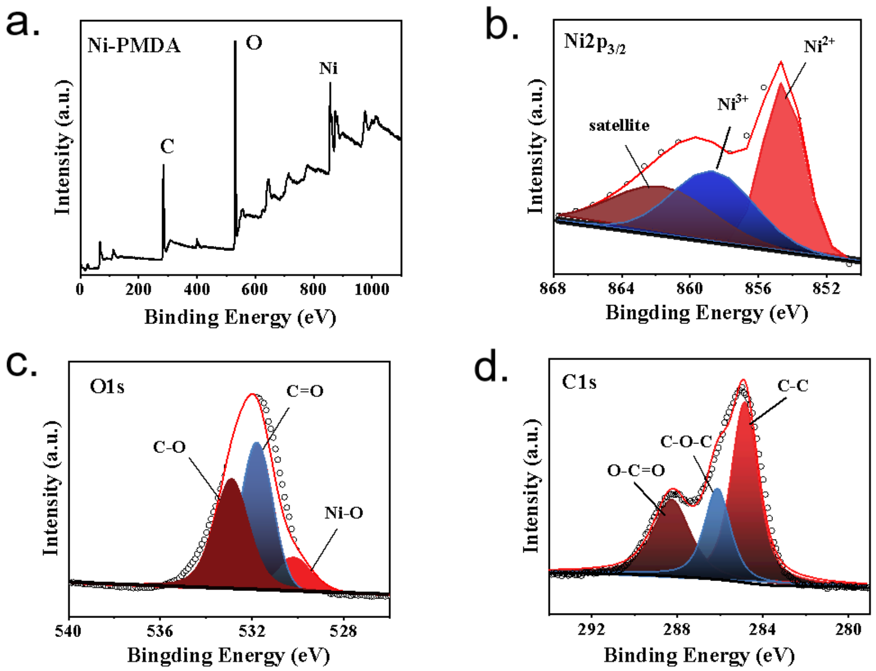

Figure S5 (a) XPS survey spectra of Ni-PMDA. (b) high-resolution $Ni2p_{3/2}$ spectrum of Ni-PMDA. (c) high-resolution O1s spectrum of Ni-PMDA. (d) high-resolution C1s spectrum of Ni-PMDA.

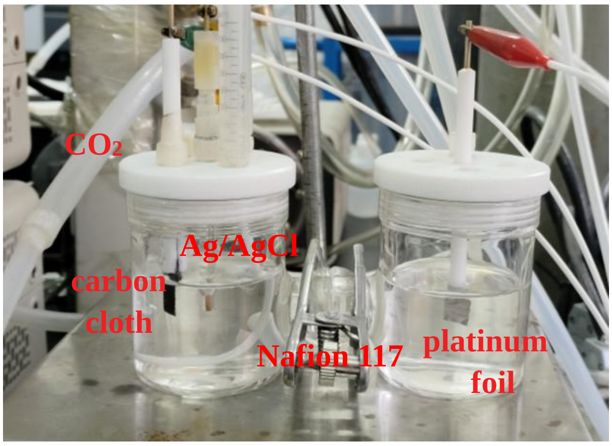

Figure S6 the experimental setup of this work

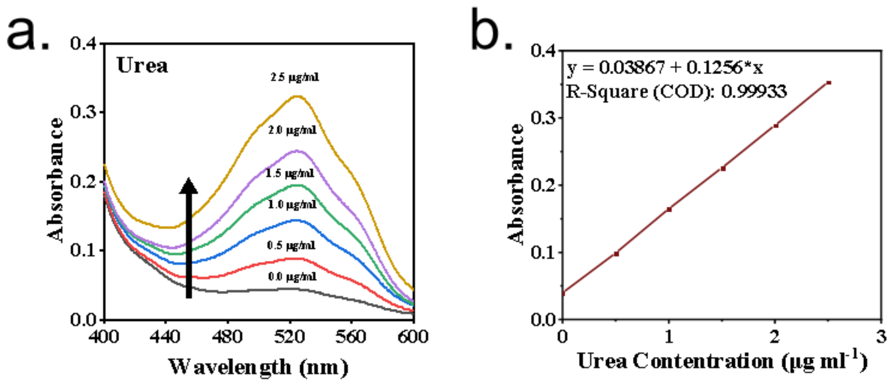

Figure S6 (a) UV-vis absorbance value at 525 nm for urea and (b) standard absorption curve.

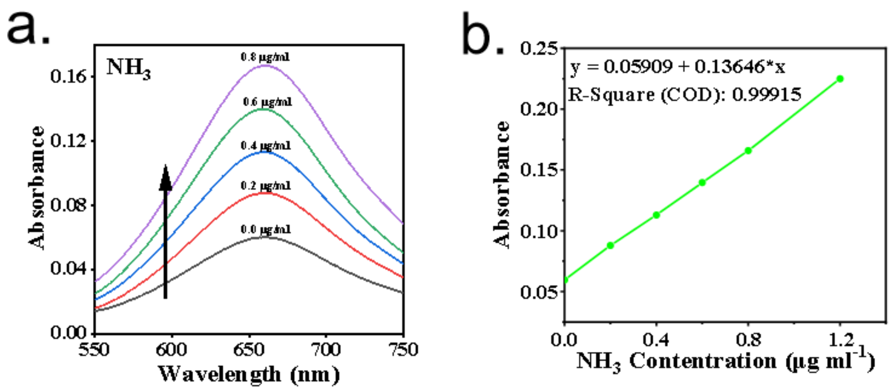

Figure S7 (a) UV-vis absorbance value at 525 nm for NH_3 and (b) standard absorption curve.

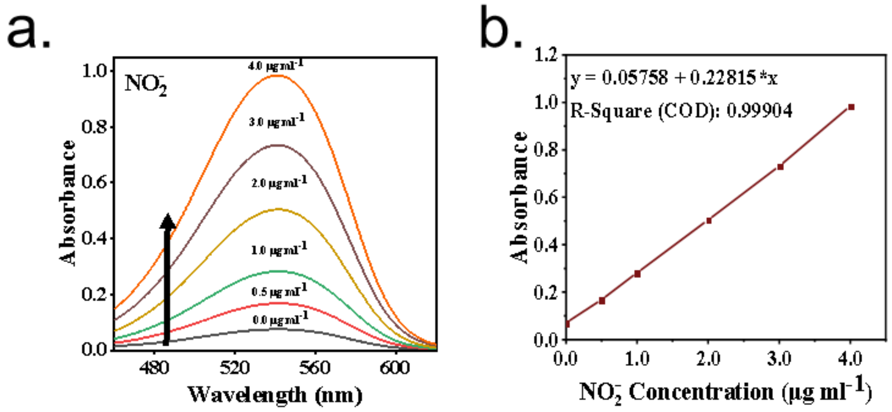

Figure S8 (a) UV-vis absorbance value at 525 nm for NO_2^- and (b) standard absorption curve.

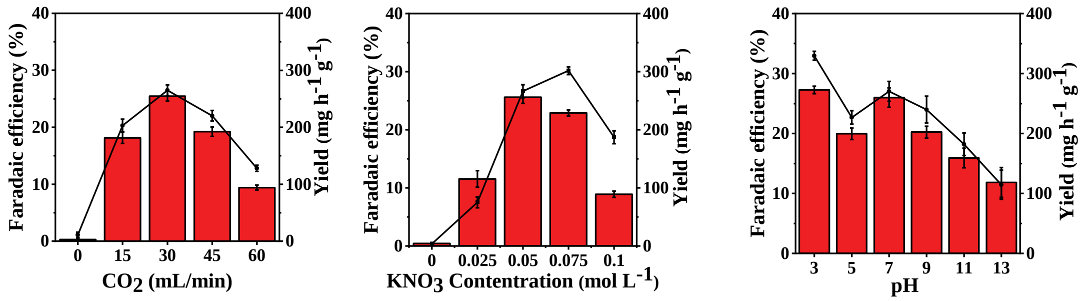

Figure S9 the urea production yield and Faradaic efficiency measured under different the electrolytic reaction conditions.

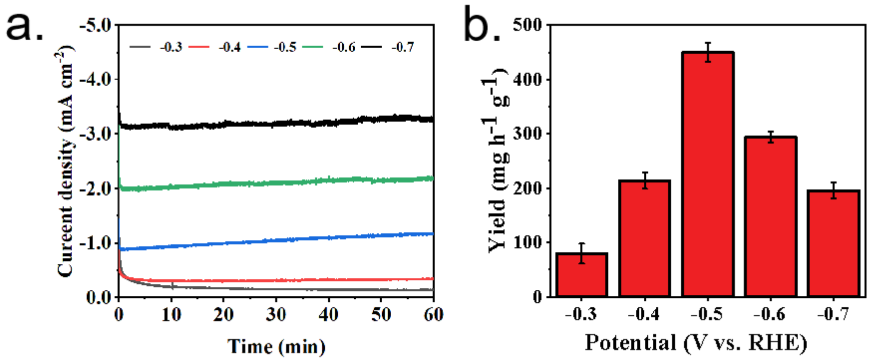

Figure S10 (a) Current density profiles of Ni-PMDA@Fe at various applied potentials. (b) Urea yield rates of Ni-PMDA@Fe under different electrocatalytic potentials.

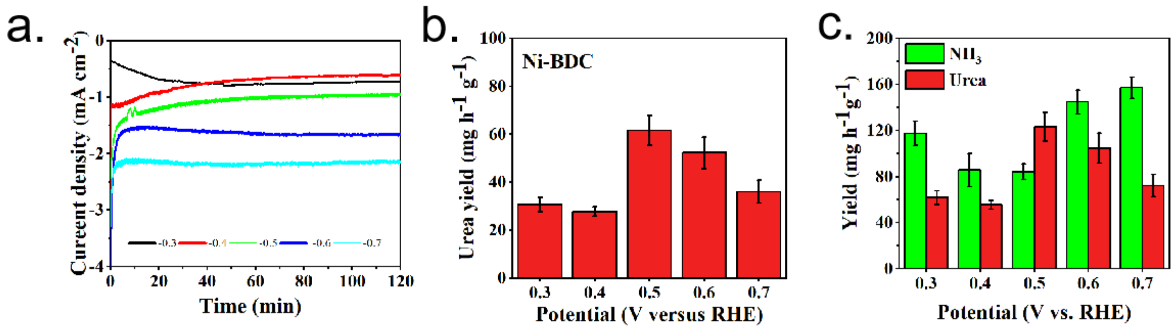

Figure S11 (a) Current density curves and (b) urea yield of Ni-BDC catalysts at different potentials; (c) average urea and NH_3 yield of Ni-BDC.

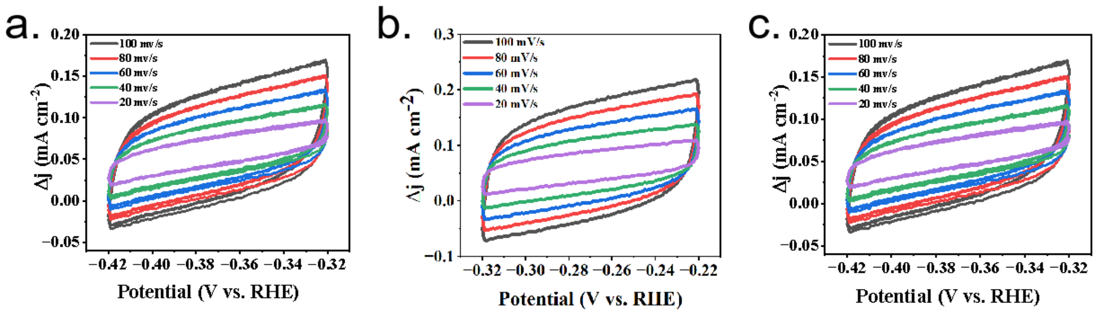

Figure S12 (a) cylic voltammetry curves of Ni-PMDA@Fe at different scan rates. (b) cylic voltammetry curves of Ni-PMDA at different scan rates. (c) cylic voltammetry curves of Ni-BDC at different scan rates.

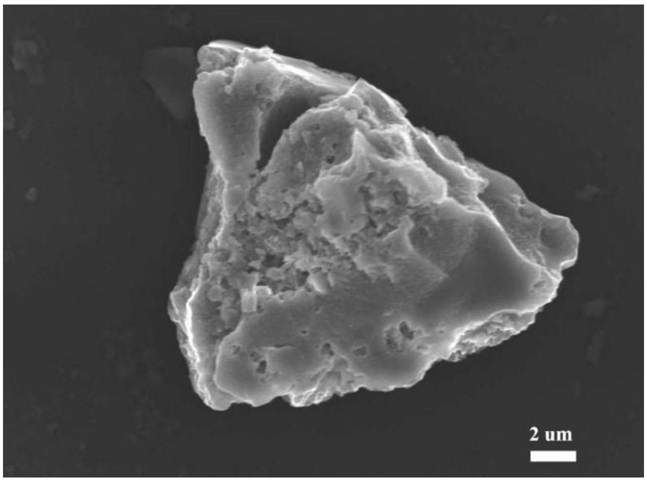

Figure S13 SEM image of Ni-PMDA@Fe after electrolysis.

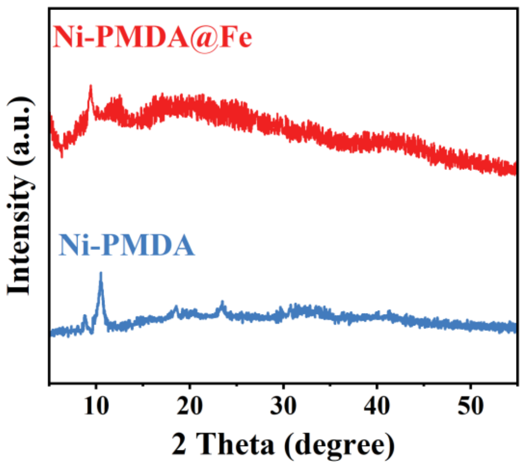

Figure S14 XRD patterns of Ni-PMDA@Fe after electrolysis.

Table S1. Recent Advances in Electrochemical Synthesis of Urea (H-cell).

Catalyst	*Potential (V_{RHE})*	*FE (%)*	*Yield Rate*	*Reference*
m-Cu_2O	-1.3	9.43	29.2 mmol•h^{-1}•g^{-1}	S1
Cu-GS-800	-0.9	28	1840 mg•h^{-1}•g^{-1}	S2
$CuWO_4$	-0.2	70.1	98.5 mg•h^{-1}•g^{-1}	S3
Pd-Cu/CBC	-0.4	69.1	763.8 mg•h^{-1}•g^{-1}	S4
Cu@Zn NW	-1.02	9.28	7.29 μmol•h^{-1}•cm^{-2}	S5
RhCu NS	-0.6	34.82	26.81 mmol•h^{-1}•g^{-1}	S6
$Cu_{97}In_3$-C	-1.4	~5	13.1 mmol•h^{-1}•g^{-1}	S7
Cu_1-CeO_2	-1.6	~5.5	52.84 mmol•h^{-1}•g^{-1}	S8
MoO_x/C	-0.6	27.7	1431.5 mg•h^{-1}•g^{-1}	S9
Fe(a)@C-Fe_3O_4/CNTs	-0.65	16.5	1341.3 mg•h^{-1}•g^{-1}	S10
$In(OH)_3$-S	-0.6	53.4	533.1 mg•h^{-1}•g^{-1}	S11
V_O-InOOH	-0.5	51	592.5 mg•h^{-1}•g^{-1}	S12
V_O-CeO_2-750	-1.6	~3.8	943.6 mg•h^{-1}•g^{-1}	S13
Diatomic Fe-Ni	-1.5	17.8	20.2 mmol•h^{-1}•g^{-1}	S14
F-CNT-300	-0.65	18	6.36 mmol•h^{-1}•g^{-1}	S15
Co_1-TiO_2	-0.8	36.2	212.8 mmol•h^{-1}•g^{-1}	S16
NC	-0.5	62	591.1 mg•h^{-1}•g^{-1}	S17
Cu_1-MoS_2	-0.4	57.02	23.3 mmol•h^{-1}•g^{-1}	S18
Cu_1Ru	-0.6	51.27	21.04 mmol•h^{-1}•g^{-1}	S19
Ni-PMDA@Fe	-0.5	41.06	449.56 mg•h^{-1}•g^{-1}	This work

Table S2. The detailed process of technical and economic assessment

Name	Symbol	Value	Unit	Note
Electrode area per fin	A-fin	2800	cm^2/fin	40 cm x 35 cm x 2
Number of electrode fins	n	12	fin/cell	
Total area of electrode	A-cell	33600	cm^2/cell	
Current density	i	25	mA/cm^2	
Total current	I-cell	8400	A/cell	
Number of cells per pack	m	1		
Operation temperature	T	25	ºC	
Cell voltage	E	2.70	V	
Power of electrolysis	P-electrolysis	2.268	kW	
Daily operation time	t	24	h	
Coulombic efficiency	CE	25	%	
Daily production rate of Urea in mole	r-mol-d	0.109	kmol-Urea/day	
Daily production rate of Urea in mass	r-mass-d	6.54	kg-Urea/day	
Daily electrolysis energy	Ee	54.432	kWh/day	
Energy intensity from electrolysis		499.37	kWh/kmol-Urea	
Electricity cost		0.09	\$/kWh	
OPEX-electrolysis	OPEX-el	44.94	\$/kmol-Urea	
Density	ρ	1.51	g/cm^3	ρ = 1.61061 - 0.0008*T
Viscosity	μ	1.85	mPa.s	μ = 2341.3*(T*^(-1.492))
Diameter of impeller	di	15.00	cm	
Rotation frequency	rps	5.00	round/s	
Reynolds # (Re)	Re	92074		Re = di^2*rps*d/μ, Re < 10
Froude # (Fr)	Fr	0.3823		Fr = ρ*rps^2/g, g = 9.81 m/s2
Power #, P0	P0	6		P0 = 6, when Re > 6,000
Power of stirring	P-stirring	0.086	kW	P = P0*di^5*rps^3*ρ

Name	Symbol	Value	Unit	Note
Daily stirring energy	Es	2.07	kWh	
Energy intensity from stirring		2.45	kWh/kmol-Urea	
OPEX-mixing	OPEX-s	0.17	$/kmol-Urea	
Operational Expenses (OPEX)	OPEX	45.11	$/kmol-Urea	
Name	Symbol	Value	Unit	Note
Power rating per cell	Pcell	22.77	kW	
Total area of electrode	A-cell	3.360	m^2/cell	
Price of cathode material		41.87	$/m2	
Cost of cathode materials	C-cm	140.45	$/cell	
Volume of KNO_3-$KHCO_3$ -H_2O electrolyte	Ve	100	L	50 cm x 50 cm x 40 cm
Concentration of KNO_3-$KHCO_3$-H_2O electrolyte	C	0.1	mol/L	
Weight of KNO_3		1.01	kg/cell	
Price of KNO_3		715.28	$/ton-$KNO_3$	Five-year average 2020-2024
Price of KNO_3		0.72	$/cell	
Weight of $KHCO_3$		1.012	kg/cell	
Price of $KHCO_3$		949.05	$/ton- $KHCO_3$	Five-year average 2020-2024
Cost of $KHCO_3$		0.96	$/cell	
Cost of KNO_3-$KHCO_3$ -H_2O electrolyte	C-electrolyte	23.17	$/cell	
Volume of PTFE		0.0125	m^3/cell	50 cm x 50 cm x 40 cm @ 1 cm
Cost of CO_2		32.1	$/ton	
Cost of $Ni(NO_3)_26H_2O$		31.34	$/cell	
Cost of PMDA		136.23	$/cell	
Cost of CO_2		4.94	$/kmol-Urea	
Weight of PTFE		27.5	kg/cell	Based on 2.2 g/cm3 of PTFE density
Price of cell body - PTFE		13,000	$/ton	2017 price
Cost of cell body - PTFE	C-body	357.50	$/cell	

Name	Symbol	Value	Unit	Note
Daily production rate of Urea	r-mol-d	0.109	kmol-Urea/day	
Capacity factor	CF	90	%	300 days a year
Annual production rate of Urea	r-mol-a	35.81	kmol-Urea/year	rmol-a = rmol-d*365*CF
Cost ratio of maintenance to system	γ	19	%	Assuming 19% fo total capital cost
Cost of total maintenance	Cmain-El	592.90	$	Replacement/repair of some electrodes & parts
Total present value of cost (TPVC)	TPVC	3749.07	$	TPVC = Ccap-ED + Cmain-ED
Time of service	t	20	year	
Annual discount rate	α	3	%	3% for real and 2.5% for nominal
Capital recovery factor (CRF)	CRF	0.0672		CRF = m*(1 + α)^m/((1 + α)^m - 1)
Equal annual cost (EAC)	ECA	251.93	$	ECA = TPVC*CRF
Levelized Capital Cost (LCC)	LCC	6.33	$/kmol-Urea	LCC = ECA/rmol-a
Levelized Total Cost (LTC)	LTC	51.44	$/kmol-Urea	LTC=LCC+OPEX

Reference:

[S1] M. Qiu, X. Zhu, S. Bo, K. Cheng, N. He, K. Gu, D. Song, C. Chen, X. Wei, D. Wang, "Boosting electrocatalytic urea production via promoting asymmetric C-N coupling" *CCS Chem.* **2023**, *5*, 2617

[S2] J. Leverett, T. Tran-Phu, J.A. Yuwono, P. Kumar, C. Kim, Q. Zhai, C. Han, J. Qu, J. Cairney, A. N. Simonov, "Tuning the coordination structure of Cu-N-C single atom catalysts for simultaneous electrochemical reduction of CO_2 and NO_3^- to urea" *Adv. Energy Mater.* **2022**, *12*, 2201500

[S3] Y. Zhao, Y. Ding, W. Li, C. Liu, Y. Li, Z. Zhao, Y. Shan, F. Li, L. Sun, F. Li, "Efficient urea electrosynthesis from carbon dioxide and nitrate via alternating Cu-W bimetallic C-N coupling sites" *Nat. Commun.* **2023**, *14*, 4491.

[S4] S. Zhang, J. Geng, Z. Zhao, M. Jin, W. Li, Y. Ye, K. Li, G. Wang, Y. Zhang, H. Yin, "High-efficiency electrosynthesis of urea over bacterial cellulose regulated Pd-Cu bimetallic catalyst" *EES Catal.* **2023**, *1*, 45.

[S5] N. Meng, X. Ma, C. Wang, Y. Wang, R. Yang, J. Shao, Y. Huang, Y. Xu, B. Zhang, Y. Yu, "Oxide-derived core-shell Cu@Zn nanowires for urea electrosynthesis from carbon dioxide an nitrate in water" *ACS Nano* **2022**, *16*, 9095.

[S6] S. Fu, K. Chu, M. Guo, Z. Wu, Y. Wang, J. Yang, F. Lai, T. Liu, "Ultrasonic-assisted hydrothermal synthesis of RhCu alloy nanospheres for electrocatalytic urea production" *Chem. Commun.* **2023**, *59*, 4344.

[S7] Y. Liu, X. Tu, X. Wei, D. Wang, X. Zhang, W. Chen, C. Chen, S. Wang, "C-bound or O-bound surface: which one boosts electrocatalytic urea synthesis" *Angew. Chem. Int. Ed.* **2023**, *62*, e202300387.

[S8] X. Wei, Y. Liu, X. Zhu, S. Bo, L. Xiao, C. Chen, T. T. T. Nga, Y. He, M. Qiu, C. Xie, D. Wang, Q. Liu, F. Dong, C.-L. Dong, X.-Z. Fu, S. Wang, "Dynamic reconstitution between copper single atoms and clusters for electrocatalytic urea synthesis" *Adv. Mater.* **2023**, *35*, 2300020.

[S9] M. Sun, G. Wu, J. Jiang, Y. Yang, A. Du, L. Dai, X. Mao, Q. Qin, "Carbon-anchored molybdenum oxide nanoclusters as efficient catalysts for the electrosynthesis of ammonia and urea" *Angew. Chem. Int. Ed.* **2023**, *62*, e202301957.

[S10] J. Geng, S. Ji, M. Jin, C. Zhang, M. Xu, G. Wang, C. Liang, H. Zhang, "Ambient electrosynthesis of urea with nitrate and carbon dioxide over iron-based dual-sites" *Angew. Chem. Int. Ed.* **2023**, *62*, e202210958.

[S11] C. Lv, L. Zhong, H. Liu, Z. Fang, C. Yan, M. Chen, Y. Kong, C. Lee, D. Liu, S. Li, "Selective electrocatalytic synthesis of urea with nitrate and carbon dioxide" *Nat. Sustain.* **2021**, *4*, 868

[S12] C. Lv, C. Lee, L. Zhong, H. Liu, J. Liu, L. Yang, C. Yan, W. Yu, H. H. Hong, Z. Qi, L. Song, K. P. Loh, Q. Yan, G. Yu, "A defect engineered electrocatalyst that promotes high-efficiency urea synthesis under ambient conditions" *ACS Nano* **2022**, *16*, 8213.

[S13] X. Wei, X. Wen, Y. Liu, C. Chen, C. Xie, D. Wang, M. Qiu, N. He, P. Zhou, W. Chen, J. Cheng, H. Lin, J. Jia, X.-Z. Fu, S. Wang, "Oxygen vacancy-mediated selective C-N coupling toward electrocatalytic urea synthesis" *J. Am. Chem. Soc.* **2022**, *144*, 11530.

[S14] X. Zhang, X. Zhu, S. Bo, C. Chen, M. Qiu, X. Wei, N. He, C. Xie, W. Chen, J. Zheng, "Identifying and tailoring C-N coupling site for efficient urea synthesis over diatomic Fe-Ni catalyst" *Nat. Commun.* **2022**, *13*, 5337.

[S15] X. Liu, P. V. Kumar, Q. Chen, L. Zhao, F. Ye, X. Ma, D. Liu, X. Chen, L. Dai, C. Hu, "Carbon nanotubes with fluorine-rich surface as metal-free electrocatalyst for effective synthesis of urea from nitrate and CO_2" *Appl. Catal. B Environ.* **2022**, *316*, 121618.

[S16] X. Tu, X. Zhu, S. Bo, X. Zhang, R. Miao, G. Wen, C. Chen, J. Li, Y. Zhou, Q. Liu, D. Chen, H. Shao, D. Yan, Y. Li, J. Jia, S. Wang, "A universal approach for sustainable urea synthesis via intermediate assembly at the electrode/electrolyte interface" *Angew. Chem. Int. Ed.* **2024**, *62*, e202317087.

[S17] Y. Li, S. Zheng, H. Liu, Q. Xiong, H. Yi, H. Yang, Z. Mei, Q. Zhao, Z.-W. Yin, M. Huang, Y. Lin, W. Lai,

S.-X. Dou, F. Pan, S. Li, "Sequential co-reduction of nitrate and carbon dioxide enables selective urea electrosynthesis" *Nat. Commun.* **2024**, *15*, 176.
[S18] W. Du, Z. Sun, K. Chen, Y. Wei, R. Bao, K. Chu, "Synergistic Cu single atoms and MoS_2-edges for tandem electrocatalytic reduction of NO_3^- and CO_2 to urea" Adv. Energy Mater. **2024**, *14*, 2401765.
[S19] F. Wang, S. Shang, Z. Li, Z. Zhang, K. Chu, "Selective urea electrosynthesis from nitrate and CO_2 on isolated copper alloyed ruthenium" *ACS Energy Lett.* **2024**, *9*, 4624.
